# The Matron's Department

**Published:** 1905-10-07

**Authors:** 


### THE MATRON'S DEPARTMENT.

### VIII.—SERVICE.

Since the essence of modern hospital management is cleanliness, the importance of the ward-maids' and scrubbers' work is beyond dispute. The general efficiency of the building from the aseptic point of view will depend very much on the manner in which this work is carried through, and one of the matron's standing difficulties will be the organisation of this band of ignorant and irregular workers, and the maintenance among them of willing obedience to orders. There is no getting rid of the fact that if she is to succeed the matron must herself know how every detail of the work ought to be done. She must know whether the fires are clumsily laid, how much polish to give out for so many square yards of flooring, and whether the steps are being washed down properly. If she is ignorant of these things and obliged to depend on a vague impression that it "looks nice" or the reverse she will be at the mercy of her subordinates, some of whom will get good work out of the servants, while others will incessantly complain of their inefficiency. If the matron on her appointment finds herself at a loss in these domestic details, she must set herself diligently to the task of learning, nor rest content until she has mastered the best possible methods. Many excellent labour-saving appliances can now be had for institution use, and progress in domestic routine has been considerable during the last ten years. It will not do to rest contented with methods acquired in some quiet home in childhood. Much can be gained from visiting other institutions and the time thus spent will be amply repaid by increased power of control.

The matron being then in a position to know exactly what results she ought to expect, and what means she should use, must concentrate attention on so organising the work as to reduce the amount of labour to a minimum. The domestic duties of the institution fall under various headings according to its size and the character of work undertaken. The following are the main branches:—

1. Housemaids' work in the wards done by wardmaids.
2. Cleansing of floors and stairs done by scrubbers.
3. Housemaids' work in nurses' home, etc.
4. Kitchen work.
5. Laundry work.
6. Engineering department.
7. Porters' and messengers' duties.

It is probable that the matron will not be called upon to superintend all these departments, but the first five enumerated will certainly fall to her share. In considering the apportionment of the work she will probably be struck by the wide differences in her new surroundings to former conditions of household management which have come under her experience. There is no regular system as regards domestic work in English institutions. The one point of agreement is diversity of organisation. A glance at the list of wardmaids and scrubbers in various London and provincial hospitals given on pages 132-135 of "Burdett's Hospitals and Charities, 1905," will show that the amount of service required bears little correspondence to the size of the institutions. It could not for a moment be maintained that the hospitals employing most wardmaids and scrubbers were better kept than the others; it is purely a question of organising labour, complicated slightly by variations in structure.

The difficulty must be met at the outset by a system of careful calculation. Starting with the wardmaids the number of hours' service required in each ward will need working out with the aid of each sister so that a fair arrangement may be arrived at. Large wards containing from 25 to 30 beds must have each its own maid, who will have also under her care any small ward attached to it, in addition to lavatories, ward kitchen, the sister's room, etc. A list of hours apportioning approximately the time required for all duties should be worked out, one copy to be given to the maid, one retained by the sister, and a third filed away by the matron. These lists compared one with another will show at a glance when the work requires for any cause less time in one ward than in another, so that spare labour can be made available in other directions. Where the services of one wardmaid have to be divided between two or more smaller wards great care must be taken to define and strictly adhere to distinct hours for each ward.

So far it has been necessary to consider each ward as a separate household. But in dealing with the cleansing of floors, passages, and stairs, a better plan is to regard the institution as a whole, to take it floor by floor as though it were an empty building, and estimate the number of hours' service required every week to keep it in good condition. In this calculation experience must go hand in hand with theory. It is impossible for anyone to say off-hand how long it should take to wash down a stone staircase extending from the top to the bottom of the building. But a careful note may be made of the time which a good worker takes over the job, and the same plan may be applied to the scrubbing of floors until a very good working average is reached of the hours required to go through the whole institution. When that has once been done all that remains is to split the work up among the scrubbers, who may be engaged by the week, day, or hour, as is most convenient. Each scrubber should have her time-table, a copy of which should be in possession of the housekeeper (if there is one) as well as of the matron herself. It is the duty of the housekeeper to check from time to time the punctuality of the workers and make a note of any irregularity in the working out of the time-table.

The housemaids' and parlourmaids' duties in the nurses' home and in the hospital should be worked out from the beginning of the day to the end, and the same course should be adopted also for the kitchen. We quote the following example from the domestic routine of an exceptionally well-managed hospital.

*Dining-room Maid.*

6.20 a.m.—Out of bedroom.
6.40 a.m.—Nurses' breakfast on table.
7 a.m.—Clear and reset sister's table for 7.40 a.m.
8 a.m.—Kitchen breakfast.
8.30 a.m.—Clear and prepare night nurses' dinner for 9 a.m.
9.30 a.m.—Clear and wash-up. Kitchen tidy.
10 a.m.—Sweep and dust dining-room. Knives. Servants' lunch.
12 a.m. to 1.—Dinners.
1.30 p.m.—Kitchen dinner.
2.30 p.m.—Help wash-up all dinners. Clean kitchen. Fetch stores. Set teas.
2 to 3 p.m.—Door bell.
4 to 5 p.m.—Teas.
5 p.m.—Wash-up all teas and reset suppers.
7.45 p.m.—Fetch suppers.
8 to 9 p.m.—Suppers and help wash-up. Reset breakfast.
10 p.m.—Fires and lights out.

*Extra Work.*

*Monday.*—Dining-room tables and safes scrubbed.
*Tuesday.*—Dining-room floor cleaned.
*Wednesday.*—Stove, paint, and globes cleaned. Door-handles.
*Thursday.*—Silver, basement, and store cupboard cleaned.
*Friday.*—Kitchen cupboard, lavatories, and passages.
*Saturday.*—Kitchen.

*Off Duty.*

Tuesday and Friday, 5.30 p.m. Alternate Sundays, 10 to 1 p.m.; 1.30 p.m. to 10.30 p.m.; 3 p.m. to 10 p.m.

We quote one more specimen—the time-table of the forewoman scrubber:—

| Name | Work | Day | Daily Hours | Weekly Hours | Wages |
|---|---|---|---|---|---|
| Mrs. Smith, Forewoman, 6 A.M. | (Nurses' quarters.) | | | | s. d. |
| | Sweeping staircase .. .. | Daily | 1 | 6 | |
| | Sweeping corridors.. .. | ,, | ½ | 3 | |
| | Basement stove .. .. | ,, | ¼ | 1½ | |
| | Fires .. .. .. .. | ,, | ½ | 3 | |
| | Board-room grates .. .. | ,, | ⅓ | 2 | |
| | Office and dusting vestibule | ,, | 1 | 6 | |
| | Medical Staff lavatory .. | ,, | ¼ | 1½ | |
| | Lodge .. .. .. .. | ,, | ½ | 3 | |
| | Polishing corridors .. | Mon. Thurs | ½ | 1 | |
| | Cleaning Board-room .. | Tues. | | 4 | |
| | Mats .. .. .. .. | Wed. | | 1 | |
| | Scrubbing staircase.. | Wed, Sat. | 3 | 6 | |
| | Cleaning store-room .. | Thurs. | | 1½ | |
| | Stores in linen-room .. | Fri. | | 2 | |
| | Servants' hall and lavatory | Fri. | | 2 | 10 10½ at 3d. an hour |
| | Total .. .. .. | — | — | 43½ | |

In addition to these comprehensive lists the matron will need a table of times off duty for the whole domestic staff and should never, except under pressure of urgent necessity, interfere with these hours. There is considerable difficulty in attracting the right class of servants to institution work. The great compensation which it offers for its more arduous character is the regularity of the hours on leave, and, if good servants are to be retained, their rights in this particular must be considerately dealt with.

However small the household, a wages' book, after the specimen given on page 73 of the "Uniform System of Accounts,"[1] should be kept by the matron. In a large institution she may be obliged to delegate the payments to her housekeeper, but she will find it convenient occasionally to pay the wages herself by way of keeping in touch with her staff and enforcing a seasonable word of encouragement or correction.

The maintenance of an equable domestic atmosphere depends almost entirely on justice in little things. The matron has to recognise clearly and impress on her subordinates the fact that the moulding of good wardmaids and servants lies in their own hands. It is not hard work, nor strict inspection, nor even well-earned rebuke which leads to restlessness and rebellion. It is the sense which prevails in a disorganised household that blame falls capriciously, that good work passes always unnoticed, and that nothing matters very much.

[1] "The Uniform System of Accounts for Hospitals, &c." Sir Henry Burdett, K.C.B. (Scientific Press.)

